# Slaughterhouses Fungal Burden Assessment: A Contribution for the Pursuit of a Better Assessment Strategy

**DOI:** 10.3390/ijerph13030297

**Published:** 2016-03-08

**Authors:** Carla Viegas, Tiago Faria, Mateus dos Santos, Elisabete Carolino, Raquel Sabino, Anita Quintal Gomes, Susana Viegas

**Affiliations:** 1 Environment and Health Research Group, Lisbon School of Health Technology, Polytechnic Institute of Lisbon, Lisbon 1990-096, Portugal; tiagofaria@ctn.tecnico.ulisboa.pt (T.F.); mateus.santos2094@gmail.com (M.S.); etcarolino@estesl.ipl.pt (E.C.); raquelsabino@hotmail.com (R.S.); anita.gomes@estesl.ipl.pt (A.Q.G.); susana.viegas@estesl.ipl.pt (S.V.); 2 Centro de Investigação em Saúde Pública, Escola Nacional de Saúde Pública, Universidade Nova de Lisboa, Lisbon 1600-560, Portugal; 3 Mycology Laboratory, National Institute of Health Dr. Ricardo Jorge, Lisbon, Lisbon 1649-016, Portugal; 4 Institute of Molecular Medicine, Faculty of Medicine of Lisbon, Lisbon 1649-028, Portugal

**Keywords:** fungal burden, assessment strategy, slaughterhouses

## Abstract

In slaughterhouses, the biological risk is present not only from the direct or indirect contact with animal matter, but also from the exposure to bioaerosols. Fungal contamination was already reported from the floors and walls of slaughterhouses. This study intends to assess fungal contamination by cultural and molecular methods in poultry, swine/bovine and large animal slaughterhouses. Air samples were collected through an impaction method, while surface samples were collected by the swabbing method and subjected to further macro- and micro-scopic observations. In addition, we collected air samples using the impinger method in order to perform real-time quantitative PCR (qPCR) amplification of genes from specific fungal species, namely *A. flavus*, *A. fumigatus* and *A. ochraceus* complexes. Poultry and swine/bovine slaughterhouses presented each two sampling sites that surpass the guideline of 150 CFU/m^3^. *Scopulariopsis candida* was the most frequently isolated (59.5%) in poultry slaughterhouse air; *Cladosporium* sp. (45.7%) in the swine/bovine slaughterhouse; and *Penicillium* sp. (80.8%) in the large animal slaughterhouse. Molecular tools successfully amplified DNA from the *A. fumigatus* complex in six sampling sites where the presence of this fungal species was not identified by conventional methods. This study besides suggesting the indicators that are representative of harmful fungal contamination, also indicates a strategy as a protocol to ensure a proper characterization of fungal occupational exposure.

## 1. Introduction

Airborne and settled particulate material of biological origin is referred to as organic dust in the field of occupational health [1]. This organic dust is composed of non-viable particles, generated from different sources, such as feces, litter, feed and feather formation (which produces a high quantity of allergen dandruff), but also by viable particulate matter (also called bioaerosols). Bioaerosols are comprised of airborne bacteria, fungi, viruses and their by-products, endotoxins and mycotoxins [1]. Fungal spores are complex agents that may contain multiple hazardous components. Health hazards may differ across species and strains because fungi may produce different allergens and also mycotoxins, and some species can infect humans [2].

In the period of 1950–1980, several fungal species were identified as causes of hypersensitivity pneumonitis (also called allergic alveolitis) in a number of professional occupations, including farmers, malt workers and wood workers [2].

In slaughterhouses, the biological risk is present not only from the direct or indirect contact with animal matter (feces, innards, feathers), but also from the exposure to bioaerosols [3]. Moreover, in some studies, *Aspergillus*, *Penicillium*, *Cladosporium* and *Mucor* genera were isolated from the floors and walls of slaughterhouses [4,5]. In addition, ventilation systems in slaughtering and processing facilities have been identified as an additional reservoir for the aerosolization and distribution of airborne microorganisms [6]. Poultry slaughterhouses are the ones that have been most assessed regarding their bioaerosols exposure [3,6,7,8,9,10], but others have been assessed too, namely cattle, sheep and reindeer slaughterhouses [8,11].

This study intends to assess fungal contamination by cultural and molecular methods in poultry, swine/bovine and large animal (bovine and horses) slaughterhouses, more precisely in the different processing areas from each unit. Fungal burden characterization will be helpful to know the background level of fungal contamination and to identify suitable indicator parameters for these settings regarding occupational exposures.

## 2. Materials and Methods

### 2.1. Assessed Settings

Three slaughterhouses were assessed between January and June from 2015 during a normal working day. One poultry slaughterhouse, one of both a swine and a bovine slaughterhouse and one large animal slaughterhouse were selected. 

The poultry slaughterhouse (PS) is located in Coimbra district. It has 400 workers distributed by several production phases. The main activities are slaughtering (8500 chickens·h^−1^), evisceration (6000 chickens·h^−1^) and meat preparation for storage and selling. The swine/bovine slaughterhouse is located in Setubal district and it has 189 workers. The main activity is slaughtering (150 tons/day). The large animal slaughterhouse (LAS) is located in Lisbon district, and it has 31 workers. The average of animals killed per week is 280. All of the three units have Portuguese and International quality certification regarding food safety.

The sampling sites selected for each of these settings were chosen based on the high amounts of time spent by the workers in those places during their occupational activity (Table 1).

In addition to conventional methods, molecular methods were also applied to detect fungal DNA (Table 2). This approach was performed to overcome some limitations of the culture-based methods and whenever specific species/strains needed to be detected. Besides the working clothes worn in all units for hygienic purposes, only the workers from bird hanging at the poultry slaughterhouse use protective masks and protection glasses as protection devices.

### 2.2. Sample Collection

#### 2.2.1. Conventional Methodologies

Air samples were collected by the use of conventional methods (Table 2). The amount of collected air ranged from 100 L (from poultry and swine/bovine slaughterhouses) to 250 L (large animal slaughterhouse). Air samples were collected through the impaction method with a flow rate of 140 L/min onto malt extract agar (MEA) supplemented with chloramphenicol (0.05%), using the Millipore air Tester (Millipore). Samplers were placed at a height of 0.6–1.5 m above the floor, approximately at the breathing zone level, and as close as possible to the worker during a normal working day. An outdoor sample was also collected to be used as a reference. Surface samples were collected by swabbing the surfaces of the same indoor sites, using a 10 by 10 cm square stencil disinfected with 70% alcohol solution between samples according to the International Standard ISO 18593 (2004). The obtained swabs were then streaked onto MEA.

#### 2.2.2. Molecular Methodologies

Air samples of 300 L were collected using the impinger Coriolis μ air sampler (Bertin Technologies), at 300 L/min airflow rate. Samples were collected onto 10 mL of sterile phosphate-buffered saline with 0.05% Triton X-100, and the collection liquid was subsequently used for DNA extraction. 

### 2.3. Sample Preparation and Analysis

#### 2.3.1. Conventional Methodologies

All of the collected samples were incubated at 27 °C for 5–7 days. After laboratory processing and incubation of the collected samples, quantitative (colony-forming units: CFU/m^3^ and CFU/m^2^) and qualitative results were obtained, with the identification of the isolated fungal species. For species identification, microscopic mounts were performed using tease mount or Scotch tape mount and lactophenol cotton blue mount procedures. Morphological identification was achieved through macro- and micro-scopic characteristics, as noted by De Hoog *et al.* [12]

#### 2.3.2. Molecular Methodologies

Five milliliters of the collection liquid were centrifuged at 2500× *g* for 10 min; the supernatant was removed, and DNA was then extracted using the ZR Fungal/Bacterial DNA MiniPrep Kit (Zymo Research, Irvine, CA, USA) according to the manufacturer’s recommendations.

Molecular identification of the different species/strains (Table 2) was achieved by real-time PCR (RT-PCR) using the Rotor-Gene 6000 qPCR Detection System (Corbett). Reactions included 1 × iQ Supermix (Bio-Rad), 0.5 μM of each primer (Table 3) and 0.375 μM of TaqMan probe in a total volume of 20 μL. Amplification followed a three-step PCR: 40 cycles with denaturation at 95 °C for 30 s, annealing at 52 °C for 30 s and extension at 72 °C for 30 s. A non-template control was used in every PCR reaction. As positive controls for the species, DNA samples were obtained from reference strains from the Mycology Laboratory from the National Institute of Health Doutor Ricardo Jorge.

### 2.4. Data Analysis

The data analysis was performed and used descriptive statistics using frequency, median and graphical representations appropriate for the nature of the data. To compare CFU/m^3^ in air and CFU/m^2^ on surfaces between slaughterhouses (PS, SBS and LAS), the Kruskal–Wallis test was used. For the statistical analysis, we used the statistical software SPSS V21. The results are considered significant at a 5% significance level.

## 3. Results

### 3.1. Fungal Load

**Figure 1:**
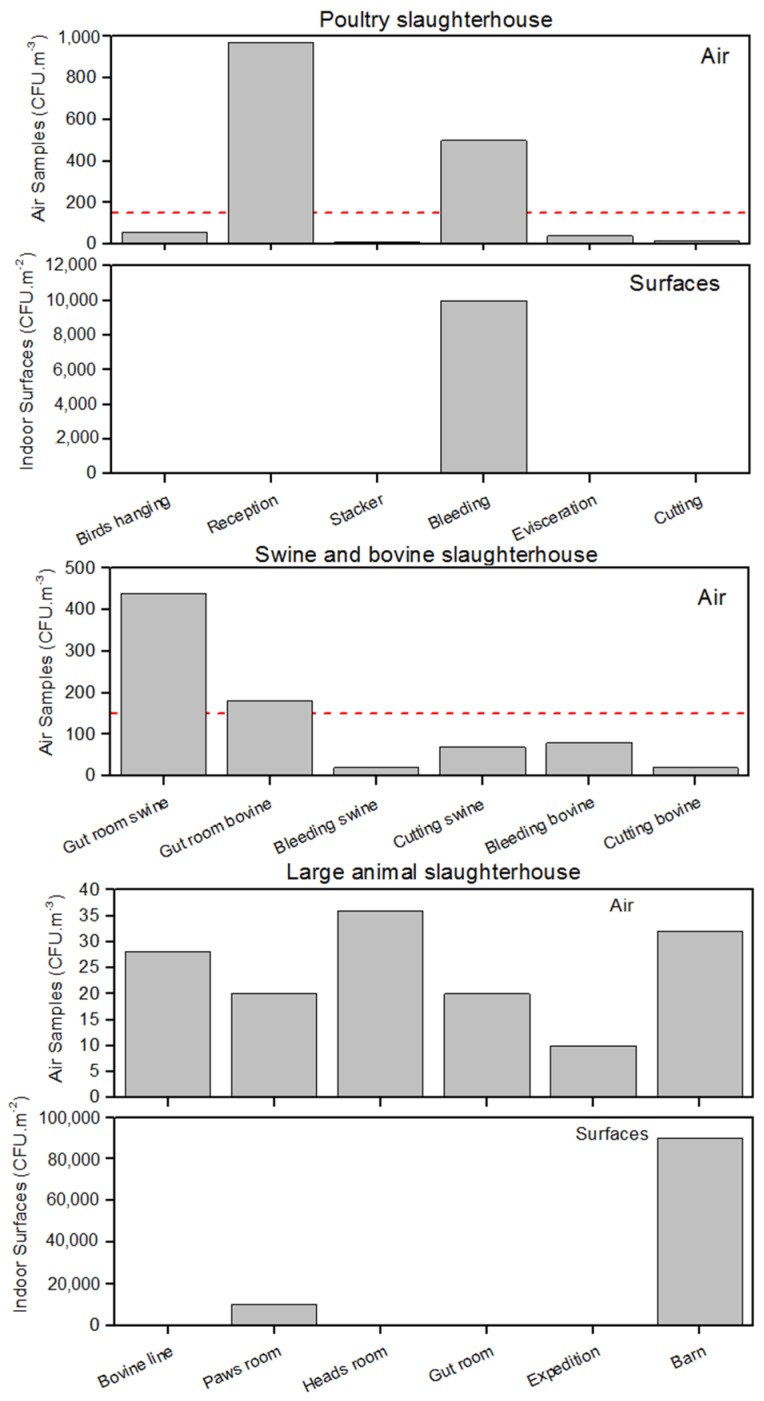
Fungal load distribution in the three assessed slaughterhouses. The dashed line represents the reference limits suggested by the World Health Organization (WHO).

Air fungal load ranged from 16 CFU/m^3^ to 970 CFU/m^3^ in the poultry slaughterhouse, 20 CFU/m^3^ to 440 CFU/m^3^ in the swine/bovine slaughterhouse and 10 CFU/m^3^ to 36 CFU/m^3^ in the large animal slaughterhouse (Figure 1). The surfaces present results that ranged from 0 CFU/m^2^ to 10,000 CFU/m^2^ in the poultry slaughterhouse and 0 CFU/m^2^ to 90,000 CFU/m^2^ in the large animal slaughterhouse. No fungal isolates were found in swine/bovine slaughterhouse surfaces (Figure 1). Poultry and swine and bovine slaughterhouses presented each two sampling sites that surpass the guideline proposed by World Health Organization (WHO) (maximum value of 150 CFU/m^3^) [13].

In the three units assessed, 11 out of the 18 samples collected presented a higher indoor fungal load when compared to the outdoor sampling, the poultry slaughterhouse being the one with the highest number of samples with increased load indoors (six out of the six samples collected). 

Comparing the CFU/m^3^ in air and the CFU/m^2^ on surfaces among the three units, no statistically-significant differences were detected.

### 3.2. Fungal Identification

#### 3.2.1. Poultry Slaughterhouse

Eight different fungal species were detected in indoor air in a total of 1596 isolates. *Scopulariopsis candida* was the most frequently-isolated species (59.5%) followed by *Penicillium* sp. (32.8%). Other fungi were also identified in this unit, namely: isolates belonging to the *Aspergillus fumigatus* complex; *Aspergillus niger* complex; *Mucor* sp.; *Geotrichum* sp.; *Paecilomyces* sp.; and *Trichoderma* sp. Only *Mucor* genera were isolated on surfaces (Table 4).

#### 3.2.2. Swine and Bovine Slaughterhouse

Seven different fungal species were detected in indoor air in a total of 810 isolates. *Cladosporium* sp. was the most frequently-isolated genus (45.7%) followed by *Penicillium* sp. (33.3%). Other fungi were also identified in this unit, namely: isolates belonging to the *Aspergillus fumigatus* complex; *Aureobasidium* sp.; *Chrysonilia* sp.; *Fusarium poae*; and *Alternaria* sp. Regarding surfaces, no filamentous fungi were isolated (Table 4).

#### 3.2.3. Large Animal Slaughterhouse

Six different fungal species were detected in indoor air in a total of 146 isolates. *Penicillium* sp. was the most frequently isolated (80.8%). Other fungi were also identified in this unit, namely: isolates from the *Aspergillus ochraceus* complex; *Aureobasidium* sp.; *Cladosporium* sp.; *Chrysonilia* sp.; and *Chrysosporium inops*. Five different fungal species were detected on surfaces in a total of 10 × 10^4^ isolates, *Scopulariopsis brumptii* being the most found (40.0%) followed by isolates belonging to the *Aspergillus terreus* complex (30.0%). Other fungi also isolated were: *Eurotium herbariorum* (teleomorph form of *Aspergillus glaucus* complex); *Acremonium* sp.; and *Chrysosporium* sp. (Table 4).

In both slaughterhouses where fungal isolates were obtained on surfaces, different species (from the ones found in air samples) were detected. In poultry slaughterhouse, *Mucor* sp. was found only on surfaces; in large animal slaughterhouse, five species were found only on surfaces (*Scopulariopsis brumptii*; *Aspergillus terreus* complex; *Eurotium herbariorum*; *Acremonium* sp.; and *Chrysosporium* sp.).

### 3.3. Fungal Detection

DNA from toxigenic strains of the *A. flavus* complex and the *A. ochraceus* complex was not amplified by qPCR. However, qPCR analysis successfully amplified DNA from the *A. fumigatus* complex in six sampling sites where the presence of this fungal species was not identified by conventional methods (Table 5). Of note, samples with lower cycle threshold (CT) values very likely exhibit higher levels of the *A. fumigatus* complex.

## 4. Discussion

This is the first Portuguese study to comprehensively assess fungal contamination by cultural methods and molecular tools in several animal slaughterhouses and in different processing areas of those slaughterhouses.

Health data under the context of bioaerosol exposure were previously analyzed [14,15,16]; on the date of these studies, the available data were not sufficient to derive exposure limits for bioaerosols [14,15,16]. To overcome this limitation, the worst case approach was used, allowing a low cost exposure assessment. This assessment strategy will prioritize interventions in the assessed setting aiming at the implementation of safety measures [17]. It is noteworthy that only short-term air sampling was performed, and variations in fungal contamination are expected. Nevertheless, the most critical scenario was selected to perform sampling, ensuring a more demanding results analyses.

Since there are no exposure limits, the guideline proposed by the World Health Organization (WHO) (maximum value of 150 CFU/m^3^) [13] was compared to the obtained fungal load background, since it is the most strict for occupational assessment purposes. Overall, the poultry slaughterhouse presented the most critical fungal load, not only indicating two sampling sites that surpass the selected guideline, but also because all air samples presented a higher indoor fungal load when compared to the outdoor sample, which could mean the existence of indoor fungal contamination sources [18]. However, we must point out that viable bioaerosol particles constitute a small percentage of the total concentration of microorganisms [19], and therefore, we should consider a bias regarding fungal burden in all units assessed.

One of the most important stages of risk control is to verify the existence of indicator species/strains that are representative of harmful fungal contamination in the analyzed setting. For that, it is important to identify what are the best indicators for slaughterhouses’ occupational environment [20]. Furthermore, a protocol with standardized methods that ensure a proper identification and detection of the selected indicators will be very helpful [20]. In this kind of occupational environment, expected to be highly contaminated, verification should be carried out applying conventional and molecular methods [21]. 

Several studies that intend to assess occupational exposure to fungi already applied these methodologies [22,23,24,25,26]. Regarding the results obtained through conventional (cultural) methods, *Penicillium* sp. was one of the most frequent in all of the assessed units (the most prevalent in LAS and the second in PS and in SBS), and among this genus, we should consider targeting some specific species/strains, such as *Penicillium polonicum* (belonging to *Penicillium aurantiogriseum* complex) with toxigenic potential [27], related to high contamination levels in feed and food [28] and already isolated in a similar occupational environment [3]. Although not being the most prevalent genus in any of the slaughterhouses analyzed, *Aspergillus* sp. was present in all units with isolates belonging to different species/complexes. The *Fumigati* section was present in PS and in SBS; the *Nigri* section only in PS; *Circumdati, Terrei* and *Aspergilli* sections were isolated only in LAS. Besides the presence of the *A. fumigatus* complex requiring implementation of corrective measures according to the American Industrial Hygiene Association (AIHA 1996) in the Field Guide for the Determination of Biological Contaminants in Environmental Samples, we should highlight that most of the species/strains isolated from *Aspergillus* sections have toxigenic potential [27].

No fungal isolates were recovered from the surfaces of SBS. Instead of the floor, walls were the surfaces sampled, since the floor was covered with animal’s blood. In the other two units, all of the fungal species/complexes isolated on surfaces were not found in air, meaning that it is crucial to collect samples from both, air and surfaces, to ensure a complete fungal contamination assessment [29]. Among the species identified exclusively on surfaces, we must point out the *Aspergillus terreus* complex that can cause invasive infections in humans [30,31]. 

The target species-complexes selected for this study should be considered as good indicators complexes/species/strains of harmful fungal contamination in this specific occupational environment. The *A. flavus* complex (toxigenic strains) produces a mycotoxin (Aflatoxin B1 (AFB1)) that is genotoxic and a potent hepatocarcinogen [32,33]. High risks of occupational exposure to this mycotoxin through inhalation were already reported [33,34,35,36,37,38]. Besides, in previous studies developed in Portuguese poultries, toxigenic strains from the *Flavi* section were detected [25], and occupational exposure of poultry slaughterhouse workers [39] and poultry workers [40] to AFB1 was already reported. The *Circumdati* section, mainly *A. ochraceus*, is known to be one of the predominant producers of ochratoxin A (OTA) detected worldwide in various food and feed sources [27]. Exposure to OTA has been related to several diseases in both animals and humans, predominantly affecting the kidney. However, besides nephrotoxicity, other toxic effects have been associated with OTA exposure, such as neurotoxicity, immunotoxicity, myelotoxicity, reproductive toxicity and teratogenicity [41,42,43]. Additionally, and since 1993, OTA has been classified as a possible carcinogen to humans in Group 2B [32], based on its known tumorigenicity in rodents. Occupational exposure to OTA has already been reported in other occupational settings, such as waste management [44], coffee production [45] and observed a high presence in the settled dust of grain elevators [46]. 

*Aspergillus fumigatus* is the species with higher clinical relevance [47,48] and the most common cause of invasive aspergillosis [49]. Moreover, one of the most abundantly-produced metabolites by this fungus is gliotoxin, which exhibits a diverse array of biologic effects on the immune system [50]. Besides, at least in two different Portuguese settings, it was proven that molecular tools increase the detection of this species-complex, namely poultries [25] and in the waste industry [51].

The use of the two types of analytical methods in this study, culture analysis and PCR-based detection, has given a more accurate scenario regarding fungal contamination in this occupational environment. This strategy allows the quantification and identification of fungal species that pose higher occupational risk due to inhalation and also the comparison of their levels with a selected guideline. In addition, with molecular tools, we can target selected species (indicators) that can be inhibited with other fungal species with fast growth rates [25,26,52]. However, the *Circumdati* section was identified in low counts in LAS, but not detected by the molecular tools applied. The same happens in one sampling site for the *Fumigati* section in SBS. False negatives are often a problem in PCR assays for microbial detection. These might be caused by several factors, including the ineffective release of microbial DNA content from the cells, poor DNA recovery after extraction and purification steps and inappropriate removal of PCR inhibitors in the analyzed samples [53]. In our study, in particular, one of the inhibition sources could potentially be the presence of particles in the air, as already observed in previous studies [54].

Prevention of fungal dissemination in slaughterhouses is of great importance in order to avoid mycotoxin production, since this can result in mycotoxin exposure for workers and, even in some cases, for consumers [55].

In order to reduce the potential exposure to fungal burden, protective measures should be taken. Preventive measures should be implemented, such as disinfection and also wearing personal protection devices, such as filtration half masks and gloves.

## 5. Conclusions

Besides the given information regarding fungal contamination background in this specific occupational environment, this study suggests the indicators that are representative of harmful fungal contamination. The applied strategy, using cultural-based methods and molecular tools in parallel, as a protocol to ensure the proper characterization of fungal contamination occupational exposure is also an added value from this assessment.

Moreover, this specific occupational environment is a good example of the reality of occupational exposures: co-exposure to several risks factors by different exposure routes. This implies the need for caution when comparing exposure results with occupational exposure limits and also for considering the cumulative risk assessment as the best option when performing risk assessment.

## Figures and Tables

**Table 1:** Sampling sites selected from each slaughterhouse.

Poultry Slaughterhouse (PS)	Swine/Bovine Slaughterhouse (SBS)	Large Animal Slaughterhouse (LAS)
Birds hanging	Gut room swine	Bovine line
Reception	Gut room bovine	Paws room
Stacker	Bleeding swine	Heads room
Bleeding	Cutting swine	Gut room
Evisceration	Bleeding bovine	Expedition
Cutting	Cutting bovine	Barn

**Table 2:** Number of samples collected and the fungal species targeted.

Slaughterhouses	Conventional Methods		Molecular Biology	Fungal Species Assessed by Molecular Biology
	Air Samples	Surface Samples	Air Samples	
Poultry	6	6 (floor)	6	*A. flavus* complex (toxigenic strains) *A. fumigatus* complex *A. ochraceus* complex
Swine	6	6 (walls)	6	
Large animal	6	6 (floor)	5	
Total of samples	18	18	17	

**Table 3:** Sequence of primers and TaqMan probes used for real-time PCR.

Fungal Species Targeted	Sequences
***A. flavus* complex (Toxigenic Strains)**	
Primer Forward	5‘-GTCCAAGCAACAGGCCAAGT-3‘
Primer Reverse	5‘-TCGTGCATGTTGGTGATGGT-3‘
Probe	5‘-TGTCTTGATCGGCGCCCG-3‘
***A. fumigatus* complex**	
Primer Forward	5‘-CGCGTCCGGTCCTCG-3‘
Primer Reverse	5‘-CGTGAGGCGGGAGCA-3‘
Probe	5‘-CCAACCTCCCACCCGTG-3‘
***A. ochraceus* complex**	
Primer Forward	5‘-CGGGTCTAATGCAGCTCCAA-3‘
Primer Reverse	5‘-CGGGCACCAATCCTTTCA-3‘
Probe	5‘-CGTCAATAAGCGCTTTT-3‘

**Table 4:** Most common fungi isolated in the three slaughterhouses.

Fungal Identification	Fungal Quantification
**Indoor air samples (PS)**	**(CFU/m^3^; %)**
*Scopulariopsis* sp.	950; 59.52
*Penicillium* sp.	524; 32.83
*Aspergillus fumigatus* complex	50; 3.13
Others	72; 4.5
**Indoor surfaces samples (PS)**	**(CFU/m^2^; %)**
*Mucor* sp.	10,000; 100
**Indoor air samples (SBS)**	**(CFU/m^3^; %)**
*Cladosporium* sp.	370; 45.7
*Penicillium* sp.	270; 33.3
*Aureobasidium* sp.	90; 11.1
Others	80; 9.9
**Indoor air samples (LAS)**	**(CFU/m^3^; %)**
*Penicillium* sp.	118; 80.8
*Aspergillus ochraceus* complex	8; 5.5
*Cladosporium* sp.	8; 5.5
Others	12; 8.2
**Indoor surfaces samples (LAS)**	**(CFU/m^2^; %)**
*Scopulariopsis brumptii*	40,000; 40
*A. terreus* complex	30,000; 30
Others	30,000; 30

**Table 5:** Conventional quantification of isolates and molecular detection from *A. fumigatus* complex in the three slaughterhouses.

Sampling Sites	Fungal Quantification		Fungal Detection
**Poultry slaughterhouse (PS)**	**Air (CFU/m^3^)**	**Surfaces (CFU/m^2^)**	**Real-time PCR (Ct, cycle threshold)**
Birds hanging	-	-	n. d.
Reception	-	-	n. d.
Stacker	10	-	35.7
Bleeding	30	-	35.92
Evisceration	10	-	36.53
Cutting	-	-	n.d.
**Swine and bovine slaughterhouse (SBS)**	**Air (CFU/m^3^)**	**Surfaces (CFU/m^2^)**	**Real-time PCR (Ct, cycle threshold)**
Gut room swine	-	-	33.91
Gut room bovine	10	-	n.d.
Bleeding swine	-	-	33.53
Cutting swine	-	-	33.61
Bleeding bovine	-	-	n.d.
Cutting bovine			35.89
**Large animal slaughterhouse (LAS)**	**Air (CFU/m^3^)**	**Surfaces (CFU/m^2^)**	**Real-time PCR (Ct, cycle threshold)**
Bovine line	-	-	n.d.
Paws room	-	-	35.85
Heads room	-	-	n. d.
Gut room	-	-	n.d.
Expedition	-	-	34.47

n.d.: not detected.
